# AI-enabled special education services: the moderating role of parental involvement in home-school-community collaboration

**DOI:** 10.3389/fpsyg.2026.1772998

**Published:** 2026-03-30

**Authors:** Xiao-Lin Liu, Tong-Tao Cao, Yang Kong, Wei-Wei Yu, Jia-Bin Qu, Feng-Jun Liu

**Affiliations:** School of Health Management, Binzhou Medical University, Yan'tai, China

**Keywords:** AI-enabled special education, inclusive education, moderating analysis, parental involvement, perceived service effectiveness

## Abstract

The implementation of inclusive education relies not only on institutional support but also on parents’ attitudinal readiness and depth of involvement. Drawing on the Technology Acceptance Model and self-efficacy theory as interpretive frameworks, this study explores the moderating role of parental involvement in the relationship between AI-enabled special education services and perceived service effectiveness. A questionnaire survey was conducted among 386 parents of children with special needs, and structural equation modeling was used to analyze the moderating effect. The results show that AI empowerment is significantly and positively associated with perceived service effectiveness (*β* = 0.31), family learning support shows a direct positive association (*β* = 0.18), while the direct effect of home-school communication is not significant. Both forms of involvement significantly moderate the relationship between AI empowerment and perceived service effectiveness, increasing the effect from *β* = 0.17–0.19 at low involvement to *β* = 0.43–0.45 at high involvement. Home-school communication functions as a pure moderator, while family learning support operates as a quasi moderator with both direct and interactive effects. The findings suggest that the association between AI services and perceived effectiveness is substantially weaker in the absence of meaningful family involvement, and that special education programs may benefit from combining technology deployment with family-centered capacity building.

## 1 Introduction

Inclusive education aims at creating a fair learning environment for children with special needs so that they can access quality education alongside other children in mainstream schools (UNESCO, 2017). Over the past decades, governments have progressively highlighted the importance of inclusive education within legal frameworks and institutional policies, considering it a matter of an educational right (Florian and Black-Hawkins, 2011). However, there are a host of challenges standing in the way of the successful execution of inclusive education. Of late, the use of artificial intelligence technology in special education is becoming increasingly pervasive, providing an avenue for meeting these challenges (Marino et al., 2023). Artificial intelligence-driven personalized learning platforms, communication aids, and intelligent assessment tools are revolutionizing special education delivery (Hussein et al., 2025), and digitally based assistive technology has shown immense potential in enhancing learning outcomes and well-being among disabled learners (Pang and Datu, 2025).

The effectiveness of special education is dependent not only on the complexity of the technology used, but, to a great extent, on the attitudes, beliefs, and knowledge of parents (Avramidis and Norwich, 2002). A meta-analysis by Goldman and Burke (2017) shows that parental involvement is a key factor in improving the effectiveness of special education services. With regard to special education supported by artificial intelligence, the perceived usefulness of the technology is a factor that directly affects parental willingness and level of involvement (Altindağ Kumaş and Sardohan Yildirim, 2024). From the home-school-community partnership outlined by Epstein (2018), efficacious parental involvement is viewed as a multiaspected concept, including home learning support, home-school communication, and community resource utilization, which cumulatively determine the overall effectiveness of the educational service rendering process. However, existing literature has given limited attention to the role of parental involvement in shaping perceptions of AI service effectiveness.

Though ample evidence supports a positive correlation between the empowerment of AI and perceived service effectiveness (Hariyanto et al., 2025; Farhah et al., 2025), the existing body of work tends to separately evaluate the performance of AI services and parental engagement, and therefore fails to explore the impact of the level of parental engagement on this correlation. Although Piccolo et al. (2024) identified parental involvement as a potentially important condition for the effectiveness of robot-mediated interventions, their systematic review found insufficient evidence to empirically confirm this role. Jang (2023) conducted a meta-analysis confirming the effectiveness of parent education programs for children with disabilities, yet did not examine how parental involvement interacts with technology-specific factors. Guo and Keles (2025) found that intervention effectiveness varies depending on the type and intensity of parental engagement, but their systematic review did not model parental involvement as a moderating condition. These studies collectively indicate that the moderating function of parental involvement within AI-assisted special education contexts remains empirically underexplored. This study aims to determine whether parental involvement moderates the link between AI-enhanced special education services and perceived service effectiveness.

This study addresses three research questions: RQ1: To what extent is there a relationship between AI-enabled special education services and perceived service effectiveness? RQ2: Is there a correlation between parental involvement (home learning support/home-school communication) and perceived service effectiveness? RQ3: Does parental involvement moderate the relationship between AI empowerment and perceived service effectiveness?

## 2 The theoretical basis for AI-enabled and parental participation in improving the effectiveness of special education services

Understanding the joint influence of AI empowerment and parental engagement on perceived service effectiveness requires integrating technology acceptance and social cognition theories. Davis (1989) Technology Acceptance Model (TAM) emphasizes that perceived usefulness and perceived ease of use are core factors determining technology adoption. From empirical evidence, the Technology Acceptance Theory is an adequate conceptual framework for explaining technology acceptance in educational contexts (Scherer et al., 2019), and can be used for explaining acceptance by parents of AI support in special education.

According to Bandura (1997), self-efficacy can be conceptualized as an underlying psychological concept for understanding parental involvement. The theory identifies four sources of efficacy judgments—mastery experiences, vicarious experiences, social persuasion, and physiological states—with mastery experiences being the most influential. There is evidence from research proving a strong positive link between self-efficacy and attitudes toward inclusive education (Yada, 2022; Mudhar et al., 2024), showing that people with a strong level of self-efficacy can better support others. Altindağ Kumaş and Sardohan Yildirim (2024) showed that parents with a strong level of self-efficacy can better use technological tools for the education of their children.

Parental attitudes, a key factor influencing perceived service effectiveness, include the affective component of attitudes (feelings about inclusive education), the cognitive component of attitudes (beliefs about the efficiency of AI services), and the behavioral component of attitudes (willingness to be actively involved in these services). A meta-analysis by Dignath et al. (2022) identified the formation mechanisms of teachers’ beliefs about inclusive education, and although this work focused on teachers, the underlying attitude formation processes—involving cognitive, affective, and behavioral components—offer a plausible parallel framework for understanding parental attitudes in similar contexts. It is apparent from the evidence in the existing scholarly literature that those with high levels of self-efficacy tend to favor an inclusive approach, and positive experiences shape these views, creating a virtuous circle. The concept of family quality of life (Summers et al., 2005) emphasizes satisfaction with services and perceived family efficacy as key elements in determining special education service effectiveness, with strong links to technology acceptance and family well-being.

TAM provides the theoretical basis for the predictor construct, explaining how perceived usefulness and ease of use shape parents’ evaluations of AI-enabled services (Davis, 1989). Self-efficacy theory provides the interpretive framework for the moderator, explaining why active involvement—particularly through mastery experiences gained from home learning support—may amplify this association. Parental involvement thus serves as a boundary condition (Hayes, 2018) that determines the strength of the technology perception–effectiveness link, with different forms of involvement drawing on distinct efficacy sources: vicarious learning for home-school communication and mastery experience for home learning support (Bandura, 1997). It should be noted that self-efficacy and parental attitudes are invoked in this study as interpretive frameworks for understanding the mechanisms through which parental involvement may shape perceived service effectiveness, rather than as directly measured constructs in the empirical model. TAM provides the theoretical basis for the AI-enabled services construct, while Bandura (1997) self-efficacy theory offers plausible explanations for why different forms of parental involvement may differentially moderate the association between AI service perceptions and perceived effectiveness. Future research incorporating direct measures of self-efficacy and attitudinal dimensions would further elucidate these proposed mechanisms.

## 3 AI applications and parental participation in special education in the Chinese context

China’s special education policy has made significant progress during the past 10 years, with relevant policy documents specifying inclusive education within a rights-based continuum (Qu, 2024). Shen and Yin (2025) proposed “appropriate inclusion” as a localized framework emphasizing the Chinese socio-cultural context. However, despite policy progress, practical implementation still faces structural barriers and unequal resource allocation (Alduais et al., 2023). According to the Ministry of Education of the People's Republic of China (2024), 912,000 students with disabilities were enrolled in various forms of special education in 2023, with only 37.42% attending the 2,345 dedicated special education schools and the majority placed in mainstream settings through inclusive education. However, access to technology-assisted instruction remains unevenly distributed across regions (Alduais et al., 2023), with rural and economically underdeveloped areas facing greater barriers to AI-enabled educational services.

The use of artificial intelligence technologies in China’s special education area is rapidly expanding and covers personalized learning tools, communication assistance tools, and intelligent assessment instruments (Hariyanto et al., 2025; Mukhtarkyzy et al., 2025). There is empirical support for the beneficial effects of technological interventions on children with disabilities, with a visible level of effectiveness for those with autism spectrum disorder (Xu et al., 2026; Atturu et al., 2025). Technological interventions in these areas provide technological assistance for the modernization of special education in China.

Parental involvement in the Chinese setting has some culturally specific features and related challenges. Although Chinese parents of children with special needs generally hold favorable views toward educational involvement, a considerable gap exists between these views and actual levels of parental engagement (Alduais et al., 2023). Certain aspects of Chinese culture, including concepts of face saving, a collectivist mind-set, and an authoritative view of professionals, may impact the parental way of communicating with school professionals. Moreover, resource constraints can be a significant drawback in facilitating parental involvement, especially given the Chinese Dual Structure of Urban and Rural Regions (Johnston and Burke, 2024).

Existing research tends to study attitudes, efficacy beliefs, and participation experiences independently, with little empirical exploration with regard to the moderating role of parental participation in the efficacy–attitudes dynamic. Jang (2023) meta-analysis confirmed the effectiveness of parent education for children with disabilities, but failed to reveal how parental participation interacts with technological factors. Ranta et al. (2025) systematic review of psychological interventions for parents of children with intellectual disabilities showed that parental participation has a positive impact on both children’s behavioral outcomes and parental well-being, but this impact may vary depending on the context of technology use. Parents of children with special needs represent an important study population because their attitudes and efficacy beliefs are actively forming and may be shaped by educational interventions, providing an opportunity to examine the proposed effects.

## 4 The moderating role of parental involvement

Parental involvement is a key moderating factor within the relationship between AI empowerment and perceived service effectiveness. Based on the theoretical frameworks discussed in Section 2, direct involvement may offer potential for enhancing mastery experiences and reducing uncertainties and may serve to strengthen the positive impact of AI empowerment on perceived service effectiveness. Bradshaw et al. (2022) pointed out that parental involvement is not only a component of the intervention but also a key moderating factor determining its effectiveness.

Empirical research supports the moderating effect of parental involvement. Aldridge et al. (2024) found a positive correlation between the level of parental participation and intervention effectiveness in technology-assisted contexts. Meta-analyses by Fang et al. (2024) and Westlake et al. (2024) demonstrated that intervention effects were more pronounced in highly involved families, suggesting that parental involvement is a contextual factor influencing the strength and direction of AI service effects.

Different levels and types of engagement may produce differentiated effects. Guo and Keles (2025) indicated that intervention effectiveness varies depending on the type and intensity of engagement. Furthermore, Hume et al. (2021) emphasized that effective parental engagement requires structured, meaningful, and long-term commitment, while Piccolo et al. (2024) highlighted the need for systematic design and ongoing support. Institutional support, professional development, and guidance play an indispensable role in this process. Alnahdi and Schwab (2024), in their research on the quality of family life for children with intellectual and developmental disabilities, demonstrated that systematic family support can significantly improve the quality and effectiveness of parental engagement.

**Figure 1:**
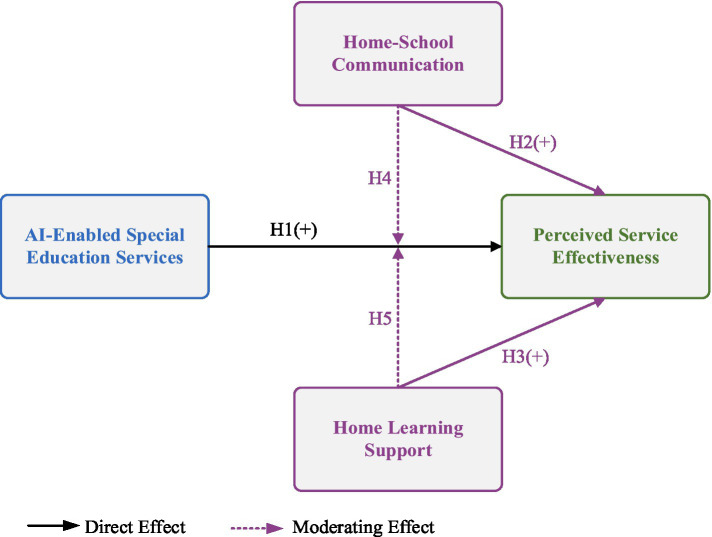
Conceptual model.

From the previous synthesis of the existing literature, it is apparent that the empowerment of AI is an important determinant of the effectiveness of services, although its effect is dependent on other factors, including parental engagement. Two common types of parental engagement include learning support in family settings and home-school communication, which can be enhancing or inhibiting factors of the effectiveness of services offered by AI entities. Therefore, this study proposes the following hypotheses (as shown in Figure 1).

*H*1: AI-empowered special education services positively predict perceived service effectiveness.

*H*2: Home-school communication involvement positively predicts perceived service effectiveness.

*H*3: Family learning support involvement positively predicts perceived service effectiveness.

*H*4: Home-school communication involvement positively moderates the predictive relationship between AI empowerment and perceived service effectiveness.

*H*5: Family learning support involvement positively moderates the predictive relationship between AI empowerment and perceived service effectiveness.

## 5 Method

### 5.1 Participants and procedure

This study included parents of children with special needs in Shandong Province whose children were receiving AI-assisted special education services. The reachable population included all parents whose children attended special education schools or inclusive education resource centers using AI-assisted teaching systems.

This study employed a combination of purposive sampling and snowball sampling. Inclusion criteria included: (1) the primary caregiver of the child with special needs; (2) the child was currently using AI-assisted educational services (such as personalized learning platforms, communication support systems, or intelligent assessment tools) for at least 3 months; and (3) the child was able to understand and complete the Chinese questionnaire. Exclusion criteria included: (1) the child only received traditional special education services and did not use AI technology; and (2) the child was not the primary caregiver. Researchers first contacted administrators of six special education schools and eight inclusive education resource centers in Shandong Province. After obtaining permission from these institutions, questionnaire invitations were sent to eligible parents through the schools. Participants were also encouraged to recommend the study to other eligible parents. While convenience sampling may limit the generalizability of the research results to other regions, this method was considered appropriate considering the research objectives and the accessibility limitations of the special needs groups.

This study was approved by the Ethics Committee of Binzhou Medical University (Approval No.: 2024-HSM-045). Informed consent was obtained electronically before participation, and no personally identifiable information was collected to ensure anonymity. The questionnaire was distributed through the Wenjuanxing platform, and data collection took place from September to November 2024.

A total of 436 eligible parents were invited to participate in the study, of whom 398 completed the questionnaire, resulting in a response rate of 91.3%. After missing data diagnosis, 12 cases were removed due to significant non-systematic missing data (missing rate exceeding 15%) or abnormal response time (less than 3 min). The final effective sample size was 386, with an effective response rate of 88.5%. Little’s MCAR test showed that the remaining missing data were completely randomized (χ^2^ = 42.67, df = 38, *p* = 0.276), and the expectation–maximization (EM) algorithm was used to imputate the small number of missing values. Before performing SEM adjustment analysis, the data were tested and confirmed to meet the assumptions of multicollinearity (VIF < 3.0), linearity, and homoscedasticity.

The demographic characteristics of the sample are detailed in Table 1. The median-split grouping reported in Table 1 was used solely for descriptive purposes and does not form part of the inferential moderation model, which treats parental involvement as a continuous variable.

**Table 1:** Demographic characteristics of the study subjects (*N* = 386).

Variable	Category	n	%
Parent gender	Female	289	74.9
	Male	97	25.1
Parent age	Under 30	47	12.2
	30–40	198	51.3
	Over 40	141	36.5
Parent education level	High school or below	126	32.6
	Bachelor’s degree	201	52.1
	Master’s degree or above	59	15.3
Child’s disability type	Intellectual disability	124	32.1
	Autism spectrum disorder	111	28.8
	Learning disability	85	22.0
	Other	66	17.1
Child’s grade level	Preschool	89	23.1
	Elementary grades 1–3	142	36.8
	Elementary grades 4–6	98	25.4
	Middle school or above	57	14.8
Duration of AI service use	Less than 1 year	123	31.9
	1–2 years	159	41.2
	More than 2 years	104	26.9
Home-school communication involvement group	High group (> Mdn)	196	50.8
	Low group (≤ Mdn)	190	49.2
Home learning support group	High group (> Mdn)	183	47.4
	Low group (≤ Mdn)	203	52.6

Parent age ranged from 26 to 58 years (M = 38.42, SD = 6.73). Mdn, Median. Median for home-school communication involvement = 3.40; Median for home learning support = 3.60. The median-split grouping was used solely for descriptive purposes; the inferential moderation model treats parental involvement as a continuous variable.

### 5.2 Measures

Data for this study were obtained using three confirmatory self-report scales widely used in special education and technology access. Scale selection was based on their proven psychometric robustness and direct relevance to the research construct. All scales underwent a standardized translation-back-translation process, and the semantic equivalence of the Chinese versions was ensured through expert review and pre-testing. The translation-back-translation process was reviewed by three bilingual experts (two in special education and one in educational measurement), and semantic equivalence was confirmed through a pilot test with 42 parents who met the inclusion criteria but were not included in the final sample. Factor validity and reliability of each scale were re-examined in this sample, confirming their applicability. All scales used a 5-point Likert scale (1 = strongly disagree, 5 = strongly agree).

The AI-enabled Special Education Services Scale adopts the perceived usefulness scale adapted from Davis (1989) technology acceptance model, and revised in combination with the application context of AI in special education. The scale contains 9 items, covering three dimensions: technology availability (3 items), service quality (3 items), and personalization (3 items). The higher the score, the more positive the perception of AI services. Example items include: “AI-assisted learning system can effectively support my child’s personalized learning needs” (personalization), “AI technology enables teachers to provide more timely feedback on children’s learning progress” (service quality), and “The AI learning tools provided by the school are easy to operate and use” (technology availability).

The Parent Engagement Scale adopted a measurement tool adapted from Epstein (2018) Parent Engagement Framework, comprising two dimensions: Home-School Communication Engagement (5 items) and Home Learning Support Engagement (5 items), for a total of 10 items. The Home-School Communication Engagement dimension measures the frequency and quality of communication between parents and the school regarding the use of AI services. Example items include: “I frequently communicate with teachers about my child’s use of AI learning tools” and “The school regularly provides me with feedback on my child’s performance in AI-assisted learning.” The Home Learning Support dimension measures the extent to which parents support and participate in their children’s AI-assisted learning in the home environment. Example items include: “I accompany and guide my child in using AI learning tools at home” and “I am able to adjust my learning support for my child based on the AI system’s recommendations.”

The perceived service effectiveness scale is adapted from the Family Quality of Life Scale by Summers et al. (2005) and the Beach Center Family-Professional Partnership Scale, and revised to incorporate the AI-assisted special education service context. The scale contains 12 items, covering three dimensions: academic progress (4 items), social adaptation (4 items), and parental satisfaction (4 items). The academic progress dimension measures parents’ perception of improved academic performance with AI assistance; example item: “AI-assisted services have significantly improved my child’s academic performance.” The social adaptation dimension measures improvements in children’s social skills and adaptive behaviors; example item: “My child’s social interaction skills have improved through AI-assisted services.” The parental satisfaction dimension measures parents’ satisfaction with the overall effectiveness of the AI service; example item: “Overall, I am satisfied with the AI-assisted special education services provided by the school.” The psychometric properties of all scales, including confirmatory factor analysis fit indices, factor loadings, and reliability coefficients, are summarized in Table 2.

**Table 2:** Summary of scale psychometric properties.

Scale/Dimension	χ^2^ (df)	CFI	TLI	RMSEA [90% CI]	SRMR	Factor loadings	*α*	ω	CR	AVE
AI-enabled services	52.85 (24)***	0.967	0.951	0.056 [0.035, 0.076]	0.038	0.68–0.84	0.891	0.893	0.894	0.586
Parental involvement (2-factor)	68.25 (34)**	0.971	0.962	0.051 [0.032, 0.070]	0.042	0.65–0.81	—	—	—	—
Home-school communication	—	—	—	—	—	—	0.856	0.858	0.860	0.553
Home learning support	—	—	—	—	—	—	0.872	0.874	0.876	0.587
Perceived service effectiveness	98.76 (51)***	0.963	0.954	0.049 [0.034, 0.064]	0.041	0.67–0.86	0.917	0.919	0.920	0.561

**p < 0.01, ***p < 0.001. The correlation between the two parental involvement dimensions was *r* = 0.47.

The AI-enabled services scale captures parents’ perceptions of technological input characteristics (availability, quality, and personalization) based on Davis (1989) TAM framework, whereas the perceived service effectiveness scale assesses child-level outcomes (academic progress and social adaptation) and overall satisfaction, adapted from Summers et al. (2005). These constructs are conceptually distinct—technology input perception versus outcome evaluation—as confirmed by the discriminant validity tests reported below.

To test the discriminant validity among the three scales, an eight-factor measurement model [AI empowerment (3 dimensions), parental involvement (2 dimensions), and service effectiveness (3 dimensions)] was constructed and compared with alternative models. The eight-factor model showed a good fit (χ^2^ = 687.42, df = 406, CFI = 0.952, TLI = 0.946, RMSEA = 0.042, SRMR = 0.045) and significantly outperformed the single-factor model (Δχ^2^ = 1842.56, Δdf = 28, *p* < 0.001) and the three-factor model (Δχ^2^ = 523.18, Δdf = 25, *p* < 0.001), indicating that each construct has good discriminant validity. The Fornell-Larcker criterion further supports this conclusion, as the smallest AVE (0.553) exceeded the largest squared inter-construct correlation (0.221).

### 5.3 Analytical strategy

Data analysis first assessed Common Method Bias, a bias that occurs when variance in the data originates from the measurement instrument rather than the underlying construct, potentially skewing observed relationships. This study employed two methods for testing this bias. First, Harman’s one-way factorial test showed that when all items were limited to a single factor, that factor explained only 31.47% of the total variance, well below the critical threshold of 50%. Second, adding the Common Method factor to the measurement model resulted in only a small improvement in model fit (ΔCFI = 0.008), far below the critical value of 0.01 (Cheung and Rensvold, 2002). These results suggest that a dominant common method factor is unlikely, although statistical tests alone cannot fully rule out the influence of shared method variance. Procedural remedies employed in this study included guaranteed anonymity, separation of predictor and outcome items into different questionnaire sections, and inclusion of reverse-coded items to reduce acquiescence bias.

Next, the normality hypothesis of the main research variables was assessed by testing skewness and kurtosis statistics and Q-Q plots. The skewness of AI-enabled services was −0.34 and the kurtosis was 0.21; the skewness of perceived service effectiveness was −0.28 and the kurtosis was 0.15; the skewness of home-school communication participation was 0.18 and the kurtosis was −0.32; and the skewness of family learning support was −0.41 and the kurtosis was 0.27. All skewness and kurtosis values were within an acceptable range of ±1.5, indicating that the variables were approximately normally distributed. The visual test of the Q-Q plots further supported this hypothesis, with the data points closely aligned with the 45-degree reference line. Furthermore, the Mardia multivariate normality test showed that both multivariate skewness (b1p = 3.42, *p* = 0.067) and multivariate kurtosis (b2p = 48.73, *p* = 0.082) were insignificant, supporting the multivariate normality hypothesis.

After completing the initial tests, composite scores for AI-enabled services, parent-school communication participation, family learning support, and perceived service effectiveness were calculated by averaging the corresponding items. It should be noted that the high/low parent participation groups reported in Table 1 are based on median splitting and are only used to describe sample characteristics and test the adequacy of subgroup sample sizes; in the moderating effect analysis, parent-school communication participation and family learning support are both included in the model as continuous variables to retain complete variable information and improve statistical power. Standardized z-scores for the four variables were calculated, and two interaction terms were created for the moderating analysis: AI-enabled × parent-school communication participation (for testing H4) and AI-enabled × family learning support participation (for testing H5). Descriptive statistics (mean, standard deviation) and Pearson correlation coefficients were calculated to test binary relationships among the research variables, directly answering H1 to H3.

The moderating analysis was performed using structural equation modeling (SEM) in R (version 4.3.2) with the lavaan package (Rosseel, 2012). Robust estimates were obtained using maximum likelihood estimation combined with bootstrap standard errors and 95% confidence intervals (5,000 resamplings). This method allows for simultaneous estimation of main effects and interaction effects, while controlling for measurement errors and exhibiting robustness to non-normal distributions. The model includes the main effect path of AI empowerment on perceived service effectiveness (H1), the direct effect path of home-school communication participation on perceived service effectiveness (H2), the direct effect path of home learning support on perceived service effectiveness (H3), the path of the AI empowerment × home-school communication interaction item on perceived service effectiveness (H4), and the path of the AI empowerment × home learning support interaction item on perceived service effectiveness (H5). Data management and composite score calculation were performed using the tidyverse package, and interaction effect visualization was performed using the ggplot2 package. Latent interaction approaches such as the LMS method (Klein and Moosbrugger, 2000) were considered but not adopted, as LMS is implemented in Mplus rather than the lavaan package used in this study, and implementing multiple simultaneous latent interaction terms via product indicator methods would substantially increase model complexity. Given the high composite reliability of all scales (CR = 0.860–0.920), the attenuation bias associated with composite-score interactions is expected to be modest. This methodological choice is acknowledged as a limitation; future research employing latent interaction modeling may yield more precise estimates of the moderating effects.

To explain the significant interaction effect, a simple slope analysis was performed to examine the conditional effect of AI empowerment on perceived service effectiveness at both high (mean + 1SD) and low (mean–1SD) levels of the moderating variable, following the procedure recommended by Preacher et al. (2007). The Johnson-Neyman technique was used to determine the significance region of the moderating effect. Statistical significance was determined based on a 95% bootstrap confidence interval: if the confidence interval did not contain zero, the effect was considered statistically significant. The effect size was interpreted using Keith (2013) standardized path coefficient criterion: *β* ≥ 0.05 for a small effect, *β* ≥ 0.10 for a moderate effect, and *β* ≥ 0.25 for a large effect. Furthermore, the standardized effect was converted to raw fractional changes to enhance the interpretability of the findings.

## 6 Results

Table 3 presents the means, standard deviations, and Pearson correlation coefficients for all research variables. Participants reported above-average levels across all measures, with family learning support (M = 3.61) slightly higher than home-school communication (M = 3.38).

**Table 3:** Mean, standard deviation, and correlation coefficient of the variables (*N* = 386).

Variable	*M*	*SD*	1	2	3	4
1. AI-enabled services	3.52	0.78	(0.89)			
2. Home-school communication involvement	3.38	0.82	0.35***	(0.86)		
3. Home learning support	3.61	0.76	0.38***	0.47***	(0.87)	
4. Perceived service effectiveness	3.67	0.71	0.42***	0.09	0.31***	(0.92)

*N* = 386. Values in parentheses on the diagonal are Cronbach’s α coefficients. All variables were measured using a 5-point Likert scale (1–5). *p < 0.05, **p < 0.01, ***p < 0.001.

Correlation analysis provided preliminary support for the hypotheses. AI-enabled services showed a significant positive correlation with perceived service effectiveness (*r* = 0.42, *p* < 0.001), initially supporting H1. Home learning support was also significantly correlated with perceived service effectiveness (*r* = 0.31, *p* < 0.001), supporting H3. However, home-school communication showed no significant correlation with perceived service effectiveness (*r* = 0.09, *p* > 0.05), not supporting H2; this relationship will be further examined in the moderation analysis.

### 6.1 Moderation analysis

**Figure 2:**
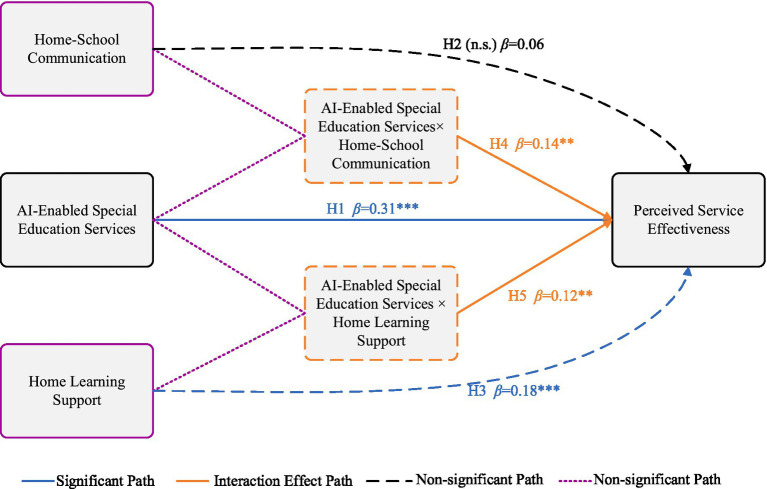
Results of structural equation modeling. ***p < 0.001; **p < 0.01; *p < 0.05; n.s., not significant. χ^2^/df = 1.69, CFI = 0.952, TLI = 0.946, RMSEA = 0.042, SRMR = 0.045.

The bootstrap standard error (5,000 samples) was used to estimate the moderated regression model to test whether the relationship between AI empowerment and perceived service effectiveness is moderated by home-school communication participation and family learning support participation. All variables were standardized before estimation, and path coefficients were interpreted using Keith (2013) effect size standards (*β* ≥ 0.05 for small effect, *β* ≥ 0.10 for medium effect, and *β* ≥ 0.25 for large effect). The results of the hypothesized moderated model are shown in Figure 2, and the hypothesis test results are summarized in Table 4.

**Table 4:** Summary of hypothesis testing results (*N* = 386).

Hypothesis	Path	β	95% CI	Result
H1	AI-enabled services → Perceived service effectiveness	0.31***	[0.22, 0.40]	Supported
H2	Home-school communication → Perceived service effectiveness	0.06	[−0.03, 0.15]	Not supported
H3	Home learning support → Perceived service effectiveness	0.18***	[0.09, 0.27]	Supported
H4	AI-enabled services × Home-school communication → Perceived service effectiveness	0.14**	[0.05, 0.23]	Supported
H5	AI-enabled services × Home learning support → Perceived service effectiveness	0.12**	[0.04, 0.20]	Supported

*p < 0.05, **p < 0.01, ***p < 0.001. 95% CIs were estimated based on 5,000 bootstrap resamples.

The analysis results show a significant positive correlation between AI empowerment and perceived service effectiveness (*β* = 0.31, 95% CI [0.22, 0.40], *p* < 0.001), supporting Hypothesis 1. This coefficient exceeds the threshold for a large effect according to Keith (2013) criteria (*β* ≥ 0.25), indicating that AI service perception has a meaningful association with perceived service effectiveness. In practical terms, for every standard deviation increase in AI service perception, the corresponding perceived service effectiveness score on the 1–5 scale increases by approximately 0.22 points (0.31 × 0.71 = 0.22), accounting for approximately 5.5% of the total scale range, indicating a moderate but meaningful improvement in effectiveness. The relationship between parent-school communication participation and perceived service effectiveness is not significant (*β* = 0.06, 95% CI [−0.03, 0.15], *p* > 0.05), and Hypothesis 2 is not supported. This result indicates that parent-school communication participation alone is insufficient to directly affect perceived service effectiveness, a finding consistent with the low binary correlation between the two variables in Table 3 (*r* = 0.09, *p* > 0.05). However, this does not mean that home-school communication is unimportant; its role may be reflected through moderating mechanisms. A significant positive correlation was found between family learning support participation and perceived service effectiveness (*β* = 0.18, 95% CI [0.09, 0.27], *p* < 0.001), supporting Hypothesis 3. This coefficient falls within the range of moderate effect sizes, indicating that direct parental involvement and support for AI-assisted learning in the home environment is associated with higher levels of perceived service effectiveness. Looking at the raw scores, for every standard deviation increase in family learning support, perceived service effectiveness increased by approximately 0.13 points, accounting for 3.2% of the total scale.

**Figure 3:**
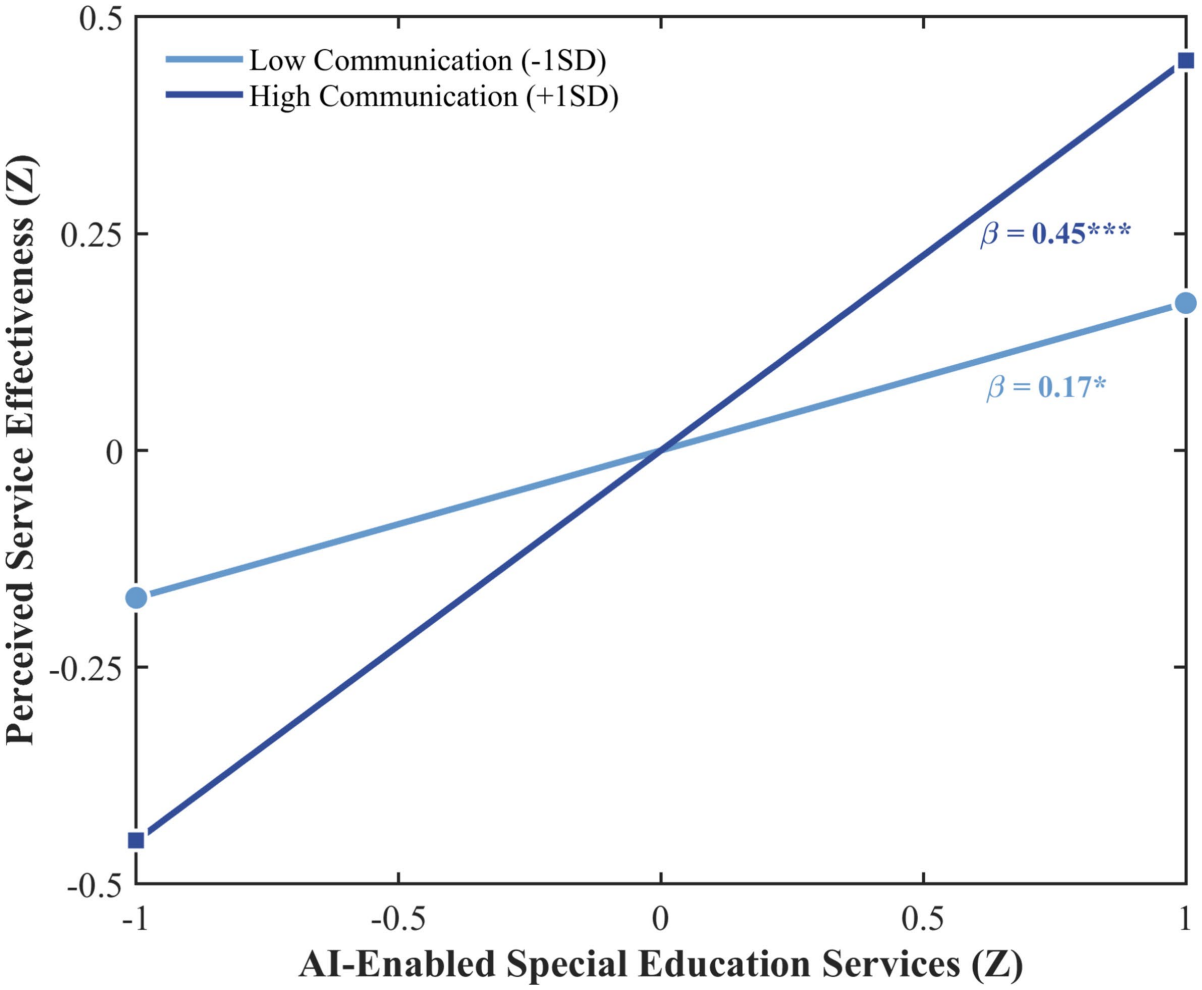
Moderating effect of home-school communication. Interaction effect *β*\beta = 0.14**, 95% CI [0.05, 0.23]. Simple slopes calculated at M ± 1SD. The inferential moderation model treats parental involvement as a continuous variable. The ±1SD grouping is used for visualization purposes only and does not reflect the analytical approach.

**Figure 4:**
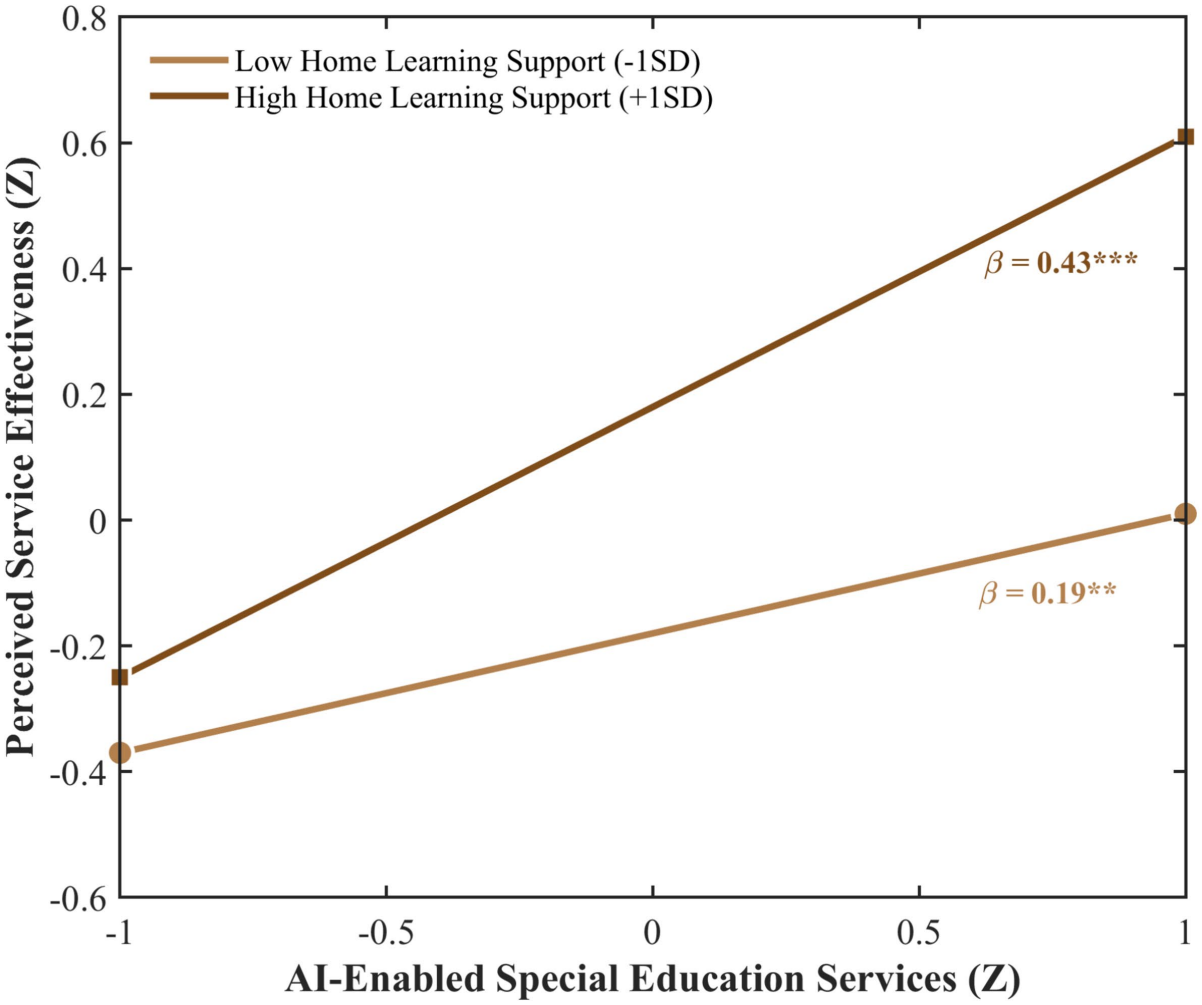
Moderating effect of home learning support. Interaction effect β\beta = 0.12**, 95% CI [0.04, 0.20]. Simple slopes calculated at M ± 1SD. The inferential moderation model treats parental involvement as a continuous variable. The ±1SD grouping is used for visualization purposes only and does not reflect the analytical approach.

Both interaction effects were significant: AI empowerment × home-school communication (*β* = 0.14, 95% CI [0.05, 0.23], *p* < 0.01) and AI empowerment × home learning support (*β* = 0.12, 95% CI [0.04, 0.20], *p* < 0.01), supporting H4 and H5, respectively. In practical terms, each standard deviation increase in home-school communication involvement increases the predictive effect of AI empowerment on perceived service effectiveness by approximately 0.10 points on the 1–5 scale (0.14 × 0.71), while each standard deviation increase in home learning support increases it by approximately 0.09 points (0.12 × 0.71). Simple slope analyses revealed that AI empowerment positively predicted service effectiveness at all levels of parental involvement, but effect strength varied substantially. At low involvement levels (M − 1SD), effects were small to moderate (home-school communication: *β* = 0.17, *p* < 0.05; home learning support: *β* = 0.19, *p* < 0.01); at high involvement levels (M + 1SD), effects reached large magnitudes (home-school communication: *β* = 0.45, *p* < 0.001; home learning support: *β* = 0.43, *p* < 0.001). As illustrated in Figures 3, 4, the slopes diverge at higher levels of AI empowerment, demonstrating the amplifying effect of parental involvement. It should be noted that the conditional effects at high levels of parental involvement (*β* = 0.43–0.45) reflect the sum of the main effect of AI empowerment (*β* = 0.31) and the interaction term evaluated at +1SD of the moderator, following the standard simple slope formula (β_conditional = β_main + β_interaction × Z; Preacher et al., 2007). The interaction effects themselves (*β* = 0.12–0.14) are of moderate magnitude according to Keith (2013) criteria, indicating that parental involvement meaningfully shapes the strength of the AI empowerment–perceived effectiveness association, though the moderating increment should be interpreted in proportion to the overall conditional effect. Notably, following the moderator typology of Sharma et al. (1981), home-school communication functions as a pure moderator, showing a significant interaction with AI empowerment despite the absence of a significant direct effect on perceived service effectiveness. In contrast, home learning support operates as a quasi moderator, contributing both directly (H3) and through moderation (H5).

In summary, four of the five hypotheses are supported (as shown in Table 4). The results reveal a significant moderating role of parental involvement in the relationship between AI empowerment and perceived service effectiveness: at high levels of parental involvement, AI empowerment has a large effect size on perceived service effectiveness (*β* = 0.43–0.45), while at low levels, the effect size is only small to moderate (*β* = 0.17–0.19). Notably, parent-school communication involvement and family learning support exhibit different mechanisms of action—the former primarily works through a moderating effect (H2 does not support it, but H4 does), while the latter has both direct and moderating effects (both H3 and H5 support it). This differentiated pattern provides empirical evidence for understanding the multifaceted mechanisms of parental involvement and lays the foundation for theoretical explanations in subsequent discussions.

## 7 Discussion

The realization of effective inclusive education requires, besides structural support and legislation, the attitudinal willingness of parents and active engagement (Woolfson, 2025). This study contributes to the literature by examining how parents’ perceptions of AI-enabled services relate to their active engagement and perceived service effectiveness. Although previous works have already verified the beneficial effect of empowerment by AI technology on the effectiveness of special education services (Marino et al., 2023; Hussein et al., 2025), the moderating effect of parental involvement has received little empirical examination. By modeling parental involvement as a moderator, this study clarifies how experiential and psychological resources shape parents’ perceptions of service effectiveness.

The significant positive effect of AI empowerment on perceived service effectiveness (*β* = 0.31) is consistent with the Technology Acceptance Model’s view concerning perceived usefulness (Davis, 1989; Scherer et al., 2019) and is consistent with findings from a meta-analysis concerning digital assistive technology use (Pang and Datu, 2025). This is significant in highlighting the need for incorporating technology acceptance experiences within special education courses.

The difference in the patterns of effects for both types of participation can be explained with the help of efficacy source theory (Bandura, 1997). Home-school communication, being an information exchange activity, relies mostly on vicarious learning and social persuasion, which is inadequate for bringing about an improvement in efficacy perceptions directly. However, family learning support may facilitate mastery experiences through direct participation, as parents who observe their children’s progress may develop stronger efficacy perceptions. A meta-analysis supports this explanation, stating the importance of contact effect quality instead of its presence (Goldman and Burke, 2017).

The moderation findings are consistent with exposure hypothesis originally identified in teacher attitude research (Avramidis and Norwich, 2002), which found that direct experience with inclusive programs was associated with more positive attitudes. Although this finding pertains to teachers, an analogous mechanism may apply to parents: sustained involvement with AI-assisted services may function as a critical condition for technology exposure to become a positive motivator. The substantial increase in effect sizes according to levels of parental involvement indicates increased importance of parental involvement in the improvement of effectiveness because of the perception of an AI service.

The two moderating mechanisms showed different patterns. According to the moderator typology proposed by Sharma et al. (1981), home-school communication functions as a pure moderator—interacting significantly with AI empowerment to shape perceived service effectiveness (H4) without exerting a significant direct effect (H2). This pattern is consistent with vicarious learning and social persuasion mechanisms (Bandura, 1997), whereby information exchange with professionals may enhance parents’ capacity to interpret and benefit from AI services, rather than independently shaping perceived effectiveness. Home learning support, by contrast, functions as a quasi moderator, contributing both a direct association with perceived service effectiveness (H3) and a significant interaction with AI empowerment (H5). This dual-pathway pattern aligns with the mastery experience mechanism, as direct involvement provides parents with tangible evidence of progress that simultaneously improves perceived effectiveness and amplifies the positive association between AI service perceptions and outcomes. Notably, even at low levels of AI empowerment, home learning support maintained a significant predictive effect (*β* = 0.19), suggesting a buffering role when technology perceptions are less favorable.

Concerning the Chinese situation, these outcomes have some specific implications. A lack of independent effect of home-school communication may be an indicator of some cultural characteristics previously discussed, such as the parents’ inclination toward professional authority, which may favor a one-way information-receiving process instead of a genuine co-partnership process (Masondo and Mabaso, 2025). Conversely, the strong effect of home learning support aligns with the traditional emphasis on family-based academic support in Chinese culture. These patterns suggest that the “appropriate inclusion” framework (Shen and Yin, 2025) should address culturally-informed strategies for enhancing parental involvement.

The theoretical contribution of this study lies in extending the technology acceptance model to the field of AI-enabled special education and integrating the moderating mechanisms of parental involvement. Bandura (1997) emphasized the central role of mastery experience in the formation of efficacy beliefs, while this study shows that AI service perception beliefs may also act as a cognitive filter, influencing how parents interpret and evaluate the significance of their involvement experiences. Parents with a strong perception of AI service will be likely to interpret involvement behaviors as a sign of increased competency, whereas those with a weak perception of AI service may likely view the same behaviors as a function of systemic dysfunction. This finding suggests that the relationship between involvement behaviors and perceived service effectiveness may be cognitively filtered through parents’ AI service perceptions, although the attitudinal mechanisms underlying this process were not directly measured in this study and warrant further empirical investigation.

Practically speaking, evidence suggests that the role of AI service initiatives must be extended beyond enabling content-driven technologies and must actively identify means of transforming parental engagement into a learning process with increased effectiveness. As home-school communication shows a substantial moderating effect but lacks a significant direct effect, educational institutions must identify new means of communicating with a greater emphasis on solving joint problems rather than transmitting information one-sidedly and enhancing the quality rather than quantity of communications. It is a priority to support capabilities related to learning within families because it is the only form of participation with a direct and moderating impact simultaneously. Parental training in the use of tools related to artificial intelligence, learning resource libraries for families, and learning models for parents and children can simultaneously increase the level and scope of participation.

From a policy standpoint, within the context of parents already engaged in AI-assisted services, the advancement of AI technology and the growth of parental capacity may need to proceed simultaneously, as technology deployment alone may not be sufficient to improve service outcomes (Alduais et al., 2023). A broad support system needs to be created, including professional counseling services, community rehabilitation centers, and special education resource centers within the community, along with effective education facilities for special needs children. The community works as an important link connecting the family and the educational institution, providing parents with an immediate avenue for professional advice and social support, thus countering the limitations imposed by the coexistence of two home-school relationships. The “appropriate inclusion” approach provides a localized framework for policymaking within a Chinese setting, with regard to the importance of considering regional disparities and the diversity of family needs with regard to resource allocation, among other issues within support systems design (Shen and Yin, 2025). Peer-learning collaborations, mentorship programs, and learning communities may further enhance efficacy expectancies and support a professional culture embracing diversity and equity.

## 8 Conclusion

This study demonstrates that AI empowerment emerged as the strongest predictor of perceived service effectiveness in this cross-sectional analysis (*β* = 0.31), while parental involvement was associated with the strength of the relationship between technological resources and perceived effectiveness. Four of the five hypotheses were supported, with family learning support having a direct effect (*β* = 0.18). Both forms of involvement significantly moderated the relationship between AI empowerment and perceived service effectiveness (home-school communication *β* = 0.14, family learning support *β* = 0.12), increasing the effect from small to moderate at low involvement (*β* = 0.17–0.19) to large at high involvement (*β* = 0.43–0.45). The two forms of involvement exhibited differentiated mechanisms: home-school communication amplified the role of AI services, while family learning support had a dual enhancing effect, contributing directly and moderating. The key implication is that the association between AI services and perceived effectiveness appears substantially weaker in the absence of meaningful family involvement. These findings suggest that special education programs may benefit from combining technology deployment with family-centered capacity building, redesigning home-school communication mechanisms, and strengthening family learning support capabilities. This study has several limitations. The sample was drawn from parents whose children were already enrolled in AI-assisted special education programs in Shandong Province, representing a population that is likely more experienced with and favorably disposed toward AI services than the broader population of parents of children with special needs. The use of purposive and snowball sampling through cooperating institutions introduces additional selection bias, as participating parents may differ from non-respondents in motivation and engagement levels. Accordingly, the findings should be interpreted as applicable to parents already engaged in AI-assisted services within this regional context, and caution is warranted in extending these results to other provinces or to parents who have not yet accessed AI-enabled education.

## Data Availability

The original contributions presented in the study are included in the article/supplementary material, further inquiries can be directed to the corresponding author.
